# Therapeutic Applications of Type 2 Diabetes Mellitus Drug Metformin in Patients with Osteoarthritis

**DOI:** 10.3390/ph14020152

**Published:** 2021-02-13

**Authors:** Parkyong Song, Ji Sun Hwang, Hyean Cheal Park, Keun Ki Kim, Hong-Joo Son, Yu-Jin Kim, Kwang Min Lee

**Affiliations:** 1 Department of Convergence Medicine, Pusan National University School of Medicine, Yangsan 50612, Korea; parkyong.song@pusan.ac.kr; 2 New Drug Development Center, Daegu-Gyeongbuk Medical Innovation Foundation, Daegu 41061, Korea; hjs1228@dgmif.re.kr; 3 Department of Life Science and Environmental Biochemistry, and Life and Industry Convergence Research Institute, Pusan National University, Miryang 50463, Korea; hcpark@pusan.ac.kr (H.C.P.); kkkim@pusan.ac.kr (K.K.K.); shjoo@pusan.ac.kr (H.-J.S.); yjkim2020@pusan.ac.kr (Y.-J.K.)

**Keywords:** osteoarthritis, type 2 diabetes mellitus, metformin, AMP-activated protein kinase

## Abstract

Type 2 diabetes mellitus (T2DM) and osteoarthritis (OA) are common chronic diseases that frequently co-exist. The link between OA and T2DM is attributed to common risk factors, including age and obesity. Several reports suggest that hyperglycemia and accumulated advanced glycosylation end-products might regulate cartilage homeostasis and contribute to the development and progression of OA. Metformin is used widely as the first-line treatment for T2DM. The drug acts by regulating glucose levels and improving insulin sensitivity. The anti-diabetic effects of metformin are mediated mainly via activation of adenosine monophosphate (AMP)-activated protein kinase (AMPK), which is an energy sensing enzyme activated directly by an increase in the AMP/ATP ratio under conditions of metabolic stress. Dysregulation of AMPK is strongly associated with development of T2DM and metabolic syndrome. In this review, we discuss common risk factors, the association between OA and T2DM, and the role of AMPK. We also address the adaptive use of metformin, a known AMPK activator, as a new drug for treatment of patients with OA and T2DM.

## 1. Introduction

Osteoarthritis (OA) and type 2 diabetes mellitus (T2DM) are common chronic diseases worldwide. About 237 million people, or 3.3% of the world’s population, suffer from OA, a condition that is much more common in older individuals [1,2]. About 10% of males and 18% of females over 60 years-of-age are affected. The main symptoms are joint pain and stiffness, caused mainly by cartilage degradation [3]. Rates of T2DM have increased markedly since 1960; indeed, 12% of adults aged 20 years and above, and 26% of those over 65, are affected [4]. T2DM is associated with long-term complications such as heart disease, stroke, blindness, and kidney failure, all of which can reduce life-expectancy by up to 10 years [1,5]. Co-existence of OA and T2DM in aging people is common, and those with T2DM seem to be more susceptible to developing OA. Piva, S.R. et al., 2015 study in adults aged 18–64 years showed that the prevalence of arthritis in those with T2DM was 52%, whereas as that in those without T2DM was 27% [6]. Although the molecular mechanism(s) underlying the high prevalence of OA in those with T2DM is not clear, OA and T2DM share risk factors such as aging and obesity. Metformin is the first-line mediation treatment for T2DM. The anti-diabetic effects of this drug are mediated via activation of AMP-activated protein kinase (AMPK) due to blockade of the mitochondria respiratory chain, resulting in an increased AMP/ATP ratio [7,8]. Since the incidence of OA in patients with T2DM increases with age, development of the effective medications for the prevention and treatment of the two diseases is necessary. In this review, we discuss common risk factors for OA and T2DM, along with the molecular mechanism(s). We also discuss preclinical/clinical application of metformin in those with OA.

## 2. OA

OA is the most common degenerative joint disease, affecting an estimated 12%–15% of the global population aged 25–74 years [9]. Over 70% of the population aged over 65 show radiographic evidence of OA [6]. Progressive degeneration of articular cartilage, synovitis, subchondral bone sclerosis, and osteophyte formation are the hallmarks of osteoarthritis. Degradation of type II collagen is the key event that determines the irreversible progression of OA [10,11]. Under normal conditions, articular chondrocytes maintain a dynamic equilibrium between synthesis and degradation of extracellular matrix (ECM) components, which include collagen type II and aggrecan (ACAN), the most abundant proteoglycan (PG) in articular cartilage [12]. As OA progresses, catabolic processes are up-regulated and anabolic processes are down-regulated, leading to severe disruption of ECM equilibrium and subsequent cartilage degradation [9]. Clinically, degradation of the ECM results in gradual impairment of articular cartilage function, usually accompanied by pain and physical disability [13]. In OA, chondrocytes become activated due to exposure to an abnormal environment, which includes high-magnitude mechanical stress, high levels of inflammatory cytokines, and increased levels of breakdown products, which can exacerbate inflammatory responses [14]. Activation of oxidative stress or inflammation-induced signaling pathways may cause phenotypic shifts in cell populations, apoptosis, and aberrant expression of inflammation-related genes such as those encoding nitric oxide synthase (NOS)-2, cyclooxygenase (COX)-2, and several matrix metalloproteinases (MMPs) (e.g., MMP-13 and ADAMTS-4 and 5) [15]. Furthermore, pro-inflammatory cytokines and matrix-degrading enzymes, along with mechanical stress, may be responsible (at least in part) for the catabolic events and downregulation of anabolic signals in osteoarthritic cartilage [16,17].

## 3. T2DM

T2DM is a form of diabetes characterized by high blood sugar, insulin resistance, and a relative lack (although not complete absence) of insulin. Hyperglycemia leads to development of a metabolic syndrome (MetS) that initiates chronic inflammation in patients [18,19,20]. T2DM is caused mainly by lifestyle factors such as obesity, lack of physical activity, an unbalanced diet, stress, and genetics [21]. Between 2001 and 2009, the prevalence increased markedly worldwide, in parallel with obesity. Although it begins in middle or older age, the rates in children and adolescents in five areas of the USA were around 21% [22,23,24]. T2DM is defined as low insulin production by pancreatic β-cells, coupled with peripheral insulin resistance [25]. Insulin resistance increases levels of fatty acids in the plasma, resulting in decreased glucose transport into muscle cells and increased fat breakdown; ultimately, this causes hepatic glucose production to increase. Insulin resistance and pancreatic β-cell dysfunction must occur simultaneously for T2DM to develop [26]. Oxidative stress and mitochondrial dysfunction are considered to be causal factors of T2DM. Consequently, increased glucose levels increase production of mitochondrial reactive oxygen species (ROS), thereby triggering inducing oxidative stress, lipid peroxidation, and impaired tissue function. Furthermore, increased ROS production is associated with hyperglycemia and development of microvascular pathologies such as neuropathy, retinopathy, and nephropathy [27]. In addition, mitochondrial dysfunction is related to insulin resistance (reduced uptake and sensitivity of tissues to glucose) [28].

## 4. Common Risk Factors for OA and T2DM

**Figure 1:**
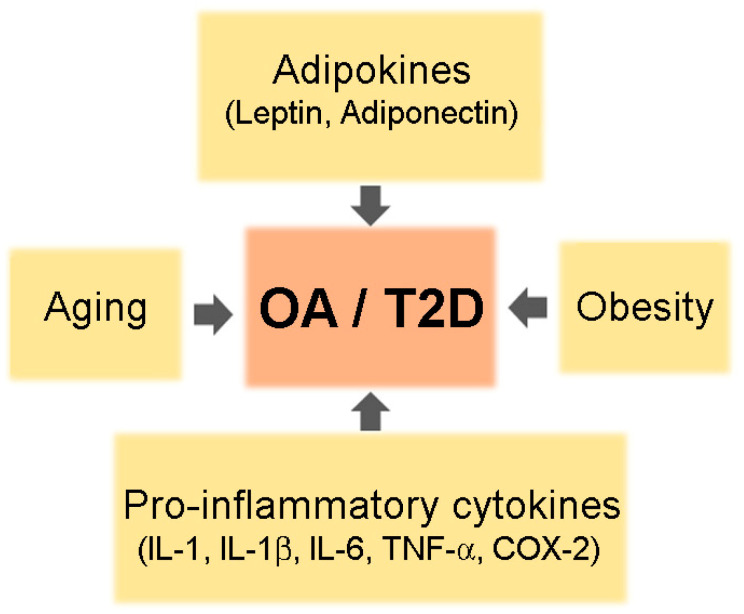
A schematic of common risk factors for osteoarthritis (OA) and type 2 diabetes mellitus (T2DM).

Co-existence of OA and T2DM in older adults is common. Although the reason for increased prevalence of arthritis in patients with T2DM is not clear, shared risk factors such as aging, obesity, and chronic inflammation may be one explanation (Figure 1).

### 4.1. Aging

Aging is one of the most common and dominant risk factors for development of OA and T2DM. In both diseases, cell function declines with age [29]. In OA, the activity of senescent chondrocytes in aging cartilage is impaired, leading to secretion of inflammatory mediators involved in cartilage degradation [30,31]. Increased accumulation of advanced glycation end-products (AGEs) and increased expression of receptor for AGE (RAGE) by aging chondrocytes alters synthetic activity and increases sensitivity to cytokines and chemokines, which trigger expression of MMPs and other inflammatory mediators [32]. Aging-related loss of autophagy (a mechanism that protects normal chondrocytes during stress responses) is associated with cell death and development of OA. In T2DM, pancreatic beta-cell activity decreases with age, as does mitochondrial health; these changes increase cartilage degradation and susceptibility to diabetes. Decreasing physical activity and nutritional deficiencies, both of which are associated with age, may also affect general health and increase the chances of developing OA and/or T2DM [33].

### 4.2. Obesity

Obesity is a well-known risk factor for initiation and progression of knee OA and T2DM [34,35]. Up to two-thirds of the elderly obese population are affected by knee OA, and over 50% of knee OA patients are obese [36,37]. For obese women, the risk of knee OA is nearly four times that of non-obese women, whereas the risk for obese men is nearly five times that for non-obese men [38]. Joints, particularly cartilage and subchondral bone tissues, are continuously exposed to mechanical stresses, an essential component of obesity-associated OA of weight-bearing joints. Increased body weight imposes greater loads on weight-bearing joints; over time this can induce wear and tear of the cartilage surface. Excess body weight is also associated with misalignment of the knee joint, which increases joint stress and promotes cartilage degradation and OA [39,40]. Excess weight can cause misalignment and increase pain in weight-bearing joints; these symptoms are strongly related to functional disability in obese people [41]. Moreover, obesity is linked to reduced strength in muscles necessary for joint stabilization; loss of muscle strength increases the load on the joint (weak muscles cannot support the joint effectively), leading to prolonged mechanical stress [42,43]. Although the detailed mechanism linking obesity to OA remains somewhat unclear, many studies indicate that mechanical and metabolic factors are the main contributors. It is estimated that about 60%–90% of all the patients with type 2 diabetes are obese (BMI ≥ 30 kg/m^2^) or overweight (30 kg/m^2^ ≥ BMI ≥ 25 kg/m^2^) [44]. Excess fat in obese people releases high amounts of fatty acids, which in turn increases insulin resistance and hyperglycemia [45,46]. Most obese individuals have elevated plasma levels of free fatty acids (FFA), which cause peripheral (muscle) insulin resistance by inhibiting insulin-stimulated glucose uptake and glycogen synthesis. FFAs also cause hepatic insulin resistance by inhibiting insulin-mediated suppression of glycogenolysis [47]. Insulin resistance and/or hypersecretion of insulin caused by obesity are thought to be important causes of T2DM.

### 4.3. Cytokines

Whereas excess weight might explain the increased risk of OA in weight­bearing joints such as the knee and hip, it is difficult to explain onset of OA in non-weight-bearing joints such as those in the hand [18]. Furthermore, a reduction in body fat is more likely to have beneficial effects on symptoms in patients with knee OA than loss of body weight [48]. This suggests that development of OA might be more complex than thought and involve both physical joint stress and systemic/non-mechanical factors. Interestingly, several reports show a link between mechanical stress and inflammation in OA; indeed, chondrocytes express mechanoreceptors at the cell surface [49,50]. Mechanical stress inhibits cartilage matrix synthesis and induces expression of pro-inflammatory factors such as interleukin (IL)-1, IL-6, tumor necrosis factor-α (TNF-α), cyclooxygenase 2 (COX-2), nitric oxide (NO), and prostaglandin E2 (PGE2) [51,52,53]. Osteoblasts in sclerotic areas of subchondral bone in OA patients show an altered phenotype, with higher expression of inflammatory mediators than osteoblasts from non-sclerotic areas. 

OA and T2DM are recognized as low grade inflammatory conditions, and the pathogenic roles for inflammatory mediators have been elucidated. IL-1, a well-studied cytokine in the context of OA, plays a prominent role in inducing expression of MMPs and other catabolic genes [51,54]. IL-1 demonstrates potent bioactivity: It suppresses synthesis of essential ECM components such as ACAN and collagen type II (COL2A1) by chondrocytes, and it promotes cartilage breakdown by inducing production of proteolytic enzymes such as MMP-1, MMP-13, and ADAMTS-4 by both chondrocytes and synovial fibroblasts [55,56]. 

IL-1β induces production of IL-6, IL-8, and leukemia-inducing factor, all of which have additive or synergistic effects on the chondrocyte catabolic cascade [57]. Consequently, pathogenic mediators such as IL-6, IL-17, TNF-α, and PGE2 stimulate production of cartilage-degrading proteases to induce ECM degradation, as well as contributing to OA-associated pain pathways [58,59]. Inhibiting synthesis of ECM components induced by inflammatory cytokines leads to release of MMPs into the joint cavity and, eventually, to cartilage degradation.

Oncostatin M acts synergistically with IL-1, IL-1β, and TNF-α to suppress expression of a number of genes associated with differentiated chondrocyte phenotypes; such genes include those encoding ACAN and COL2A1. ADAMTS-5, an aggrecanase belonging to the ADAMTS family (disintegrins and metalloproteinases with a thrombospondin-1 domain) of extracellular proteinases, is considered to be the major aggrecan-degrading enzyme involved in cartilage degradation in OA. Several studies show marked regulation of ADAMTS-4 and ADAMTS-5 after stimulation with TNF-α and oncostatin M, as well as IL-1-induced expression of ADANTS-4, by both human and mouse chondrocytes [60,61]. 

Both mechanical stress and inflammatory mediators induce NF-κB (p65/p50)- and activate mitogen-induced protein kinase (MAPK)-mediated downstream signaling pathways, which are abnormally activated in osteoarthritic chondrocytes [62]. The canonical NF-κB pathway is a central regulator of the inflammatory cytokine-induced catabolic actions of MMPs, NOS2, COX-2, and IL-1 in chondrocytes. Subsequently, released ECM components trigger inflammatory responses and induce cartilage breakdown. NF-κB signaling-mediated expression of HIF-2α up-regulates cytokine-induced expression of MMP-13 and ADAMTS-4, and RUNX2-mediated signaling regulates expression of ADAMTS-4 and ADAMTS-5 [63,64].

In addition to NF-κB pathways, mechanical and inflammatory stimuli in articular chondrocytes MAPK pathways through the ERK, c-Jun N-terminal kinase (JNK), and p38 kinase cascades. Activated transcription factors, including those of the ETS, AP-1, and C/EBP families, regulate expression of genes related to catabolic and inflammatory responses. JNK-driven activation of AP-1, MEK/ERK-induced phosphorylation of ETS factors, and p38-mediated activation of C/EBPβ and RUNX2 participate in induction of MMPs and in regulation of catabolic and inflammatory responses [65,66].

Under obese conditions, pro-inflammatory cytokines such as TNF-α, IL-6, and IL-1β activate the JNK and IKKβ/NF-κB pathways in adipocytes, hepatocytes, and associated macrophages; moreover, MCP-1 and other chemokines play essential roles in recruiting macrophages to adipose tissue [67]. Obesity-induced activation of IKKβ leads to translocation of NF-κB to cell nuclei and to increased expression of potential inflammatory mediators that promote insulin resistance and T2DM. In addition, obesity-induced activation of JNK, mediated mainly by ER stress, promotes phosphorylation of insulin receptor substrate 1 (IRS-1) at serine sites that negatively regulate normal signaling through the insulin receptor/IRS-1 axis [67,68]. In addition, JNK and IKKβ/NF-κB are activated by pattern recognition receptors such as TLRs and RAGE. Furthermore, prolonged hyperglycemia and accumulation of AGEs activates NF-κB [69]. In addition to pro-inflammatory cytokines and AGEs, cellular stressors such as ROS and ER stress activate the JNK and NF-κB pathways [70].

### 4.4. Adipokines

As obesity progresses, adipocytes release adipokines such as leptin, adiponectin, resistin, and visfatin from adipose tissue, which is considered to be a metabolic endocrine organ [71]. These adipokines contribute to the low grade inflammatory status of obese patients and affect cartilage homeostasis by inducing cartilage degradation or inflammatory responses [72,73]. The most well-known adipokine, leptin (encoded by the obese (ob) gene), was identified in 1994 as a metabolic link between obesity and OA [74]. 

Leptin levels in OA cartilage are higher than those in normal articular cartilage, and expression levels of adipokines increase upon inflammatory stimulation [75]. In addition, stimulation of articular chondrocytes with leptin, adiponectin, or resistin plus other inflammatory cytokines leads to marked induction of a diverse array of pro-inflammatory factors such as COX-2, PGE2, NOS2, IGF1, and TGFβ, as well as degenerative enzymes MMP9 and MMP13, via activation of leptin receptors and NF-κB mediated pathways [76]. In leptin-deficient (ob/ob) and leptin receptor-deficient (db/db) mice, impaired leptin signaling cannot trigger systemic inflammation and knee OA, suggesting that leptin is essential for cartilage degradation [77]. In addition, leptin regulates cartilage homeostasis by influencing osteoblast proliferation and differentiation, as well as by suppressing bone formation through a hypothalamic relay.

Another adipokine expressed in adipose tissue, adiponectin, plays a role in cartilage hemostasis by increasing expression of tissue inhibitor of metalloprotease-2 (TIMP-2), and by decreasing IL-1β-induced expression of MMP-3, in chondrocytes [78]. In addition, adiponectin up-regulates IL-10 secretion by human macrophages to increase TIMP-1 levels and prevent ECM degradation [79]. The adiponectin/leptin ratio in the synovial fluid of patients with severe knee OA is associated with reduced knee pain, indicating that adiponectin may have beneficial effects on OA [80]. However, several reports suggest that the distinct role of adiponectin is associated with radiographic severity of OA, and that it induces production of pro-inflammatory cytokines by chondrocytes [81]. The different isoforms of adiponectin may mean that research results are inconsistent; therefore, further studies are needed to identify the role of adiponectin in progression of OA. 

Elevated leptin levels are associated with insulin resistance and with development of T2DM, obesity, and hypertension. MetS is more common in T2DM patients with increased leptin levels [82,83,84,85]. Imbalanced production of adipokines by adipose tissue and other sources can aggravate insulin resistance and result in metabolic abnormalities that cause MetS. In particular, leptin plays a crucial role in the link between MetS and OA. Typical factors associated with central obesity and MetS induce pro-inflammatory macrophage polarization and activity within synovial and adipose tissue; these phenomena occur via alterations in AMPK and mTORC1 expression, as well as changes in adipokine levels. These harmful metabolic processes also affect cartilage degradation by chondrocytes.

## 5. Links between OA and T2DM

T2DM and OA are linked by the chronic systemic inflammation related to MetS [86,87]. Under hyperglycemic conditions, chondrocytes of OA patients are unable to down-regulate glucose transport into chondrocytes. Additionally, exposure to high levels of glucose induces production of ROS in OA cartilage [88]. Since the catabolic activity of ROS produces inflammatory mediators such as IL-1β and NF-κB, which promote chondrocyte degradation and apoptosis, increased ROS mediated by high glucose levels damages chondrocytes [89]. Moreover, OA chondrocytes exposed to hyperglycemic medium express higher levels of MMPs than normal chondrocytes [90]. An in vivo cohort study found that higher levels of fasting serum glucose levels are associated with greater cartilage disruption, mediated by bone marrow lesions and loss of tibial cartilage volume, in post-menopausal women than in men [91]. This sex difference might be due to reduced levels of estrogen, which plays a protective role in cartilage, after menopause. All of the findings presented in these studies demonstrate the harmful effects of hyperglycemia on articular cartilage, and suggest that altered glucose metabolism could be a direct link between OA and T2DM. 

Another pathogenic role of hyperglycemia is induction of AGEs; age-related accumulation of AGEs in articular cartilage results in a pathogenic environment and, ultimately, induces symptoms of OA such as stiffness and cartilage degradation [92,93]. High glucose concentrations in those with diabetes lead to increased formation of AGEs. AGEs and RAGE trigger the inflammatory cascade, mainly via production of pro-inflammatory TNF-α and activation of the transcription factor NF-κB [27]. These signals induce inflammation and oxidative stress, both of which promote degradation of cartilage. 

Human chondrocytes express functional insulin receptors that respond to physiologic insulin concentrations. Expression and activity of insulin receptors in OA chondrocytes is lower than that in normal chondrocytes [94]. Treatment with insulin increases expression of MMP-13 and IL-1β, and downregulates autophagy, an essential homeostatic mechanism, in chondrocytes by reducing expression of LC3 II and increasing phosphorylation of Akt and rpS6. This suggests that excess insulin, as seen in T2DM patients, may damage cartilage and cause OA [95]. Insulin is a critical negative regulator of synovial inflammation and catabolism; thus, development of insulin resistance in an obese population would diminish the ability of insulin to suppress production of inflammatory and catabolic mediators that promote OA [96].

In conclusion, low grade inflammation, oxidative stress, and dysregulation of cell function mediated by aging, obesity, and hyperglycemia are common risk factors for OA and T2DM, suggesting a strong relationship between the two diseases [97,98].

## 6. Management of OA and T2DM

This section may be divided by subheadings. It should provide a concise and precise description of the experimental results, their interpretation, as well as the experimental conclusions that can be drawn.

Recent studies demonstrate that the risk for OA in obese patients with MetS is higher than that for obese patients without MetS [99]. In addition, studies show that systemic metabolic alterations associated with T2DM occur in those with OA, suggesting that T2DM is an independent risk factor for OA development and/or severity. Understanding common risk factors for development and progression of OA and T2DM is necessary for effective diagnosis, prognosis, treatment, and prevention of these co-existing conditions.

Drug repositioning is a promising field that identifies new therapeutic opportunities for existing drugs [100]. Drug repositioning is an attractive proposition because it involves the use of de-risked compounds. Since the pharmacokinetic profiles and safety of these drugs are already established, overall development costs and timelines can be reduced markedly [101]. 

The link between OA and T2DM means drug repositioning can be used to identify new interventions. In terms of drug repositioning, well-known oral anti-diabetic drugs such as thiazolidinedione and lipid-lowering medications such as statins have been investigated as supplements to symptom­alleviating treatments for OA in OA patients with T2DM [102,103]. From this point of view, well-designed trials to test novel applications for conventional medications are forthcoming; such trials intend to confirm results from animal studies [104,105]. Progression of OA depends mainly on chronic low grade inflammation, particularly involving articular chondrocytes. AMPK plays a major role in regulating inflammatory processes; indeed, the anti-inflammatory effects of AMPK provide a strong rationale for re-examination of AMPK activators (which are already available clinically) as new mediations for OA.

### 6.1. AMPK

AMPK plays an important role in insulin signaling, whole-body energy balance, and metabolism of glucose and fats [8]. It is a key regulator of metabolism because it senses increases in the intracellular ratio of AMP and/or ADP to ATP following cellular stress, which then triggers a metabolic switch from ATP consumption to ATP generation to maintain energy balance [106,107,108]. Decreased phosphorylation of AMPKα, along with pro-catabolic responses to pro-inflammatory cytokines IL-1β and TNF-α by chondrocytes, was observed in mouse OA models and in knee cartilage from humans with OA [109,110]. These results suggest that inflammation-induced cartilage degradation could be protected by maintaining the AMPK activity [110]. In addition, AMPKα1α2 conditional double knockout (AMPKα cDKO) mice showed severe and spontaneous age-associated OA symptoms and an enhanced IL-1β-stimulated catabolic response, suggesting that AMPK activity in chondrocytes is important for maintenance of joint homeostasis [111]. Activation of AMPK by A-769662, a specific AMPK agonist, suppressed inflammatory arthritis in mouse models of antigen-induced arthritis and passive K/BxN serum-induced arthritis. Moreover, AMPK activation alleviates ER stress induced apoptosis of chondrocytes and cartilage degradation [112]. These findings suggest that specific targeting of AMPK activation may be an effective therapeutic strategy for OA [113].

Mechanistically, AMPK activation is associated with inhibition of TNF-α-mediated NF-κB signaling pathways, which are critical for pro-inflammatory effects. AMPK also downregulates the JAK/STAT signaling pathway, a crucial driver of cytokine signaling, cell growth, and apoptosis [114,115,116]. Another downstream molecule of AMPK is mammalian target of rapamycin (mTOR), which plays a major role in regulating both glucose and lipid metabolism, as well as cell growth, proliferation, and survival. In particular, mTOR inhibition by rapamycin or AMPK suppresses inflammatory diseases and osteoarthritis by regulating T-cell differentiation [117].

### 6.2. Metformin

**Figure 2:**
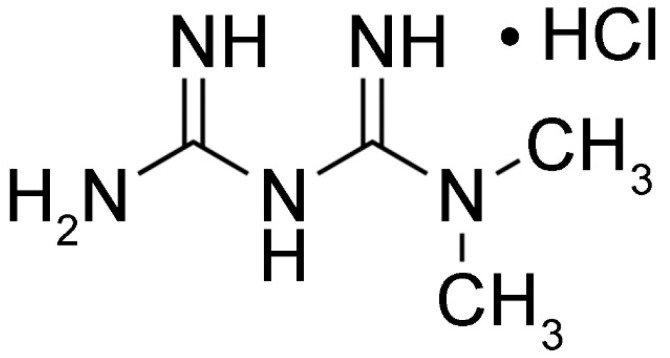
Structure of metformin.

Since diabetic patients are at higher risk of bone degradation, anti-diabetic mediations may have beneficial effects against bone disorders [7,118,119]. Metformin (Figure 2), an oral anti-hyperglycemic drug, is the first-line medication for T2DM. Metformin acts mostly by inhibiting hepatic gluconeogenesis. The main target of metformin is mitochondria, which synthesize ATP via oxidative phosphorylation, resulting in energy production [120]. ROS produced during this process can cause oxidative stress and mitochondrial dysfunction, both of which are related to insulin resistance in skeletal muscle, liver, fat, and pancreas [121,122]. Most of the metabolic effects of metformin are exerted via direct inhibition of the mitochondrial respiratory chain (complex 1), which results in ATP depletion and increased production of cytosolic AMP. Thus, activation of AMPK via phosphorylation of Thr-172 in the alpha subunit of AMPK is induced indirectly, which decreases gluconeogenesis in the liver [123]. Increased AMP levels also inhibit of adenylate cyclase, resulting in reduced production of cAMP. As a result, the activity of protein kinase A and its target, cyclic AMP response element binding protein, are inhibited; consequently, fasting glucose levels are reduced [124,125,126]. In addition to suppressing hepatic glucose production, metformin increases insulin sensitivity by inhibiting lipogenesis, increases peripheral glucose uptake by inducing phosphorylation of GLUT4 enhancer factor, and decreases insulin-induced suppression of fatty acid oxidation [127,128,129]. Moreover, metformin alleviates chronic inflammation via its anti-inflammatory activity, as well as inducing autophagy by inhibiting mTOR phosphorylation via activation of AMPK [125,130,131]. 

T2DM patients are more likely to have hand or knee OA than non-diabetic subjects. Conversely, subjects with OA have an higher risk of developing T2DM than age- and sex-matched non-OA counterparts. 

Based on the biological effects mediated by targeting the pathogenic mechanisms of OA, metformin (in addition to weight loss) might be considered as a potential disease-modifying agent for the obese phenotype of knee OA. A recent prospective cohort study reported a relationship between metformin use and reduced progression of knee OA in obese individuals; indeed, compared with non-users, those using metformin showed a decreased rate of medial cartilage volume loss over 4 years, and a trend toward a decreased risk of undergoing total knee replacement over 6 years [132]. Another nationwide, retrospective, matched-cohort study in Taiwan found that a combination COX-2 inhibitors and metformin in OA patients with T2DM was associated with lower rates of joint replacement surgery than treatment with COX-2 inhibitors alone [133]. Several animal models show the potential therapeutic effects of metformin on OA via regulation of AMPK. As demonstrated by a recent study in destabilization of the medial meniscus (DMM)-induced OA mice, both intra-gastric and intra-articular metformin treatment attenuated degradation of articular cartilage, delayed OA progression, and modulated pain-related behaviors via activation of AMPK [134]. The beneficial effect of metformin was also confirmed in genetically-modified mice. Treatment of AMPK α1 knockout (KO) and DMM-induced OA mice with metformin had no effect, suggesting that the chondroprotective effect of metformin was mediated mainly by up-regulation of AMPKα1 expression. Moreover, the study showed that oral administration of metformin effectively alleviated cartilage degradation and aging by regulating the AMPK/mTOR signaling pathway in a DMM-induced OA mouse model, which suggests that metformin administration could be an effective therapy for OA [135]. Furthermore, metformin inhibited release of NO, MMP3, and MMP13 from murine femoral head cartilage explants in response to IL-1β and TNF-α [110]. Mesenchymal stem cells (MSCs) are multipotent stromal cells that can differentiate into a variety of cell types, including osteoblasts, chondrocytes, and adipocytes; these cells can protect against cartilage breakdown by exerting immunomodulatory functions. A recent study identified the beneficial effects of metformin-stimulated adipose tissue-derived human MSCs (Ad-hMSCs) in a rat OA model. OA rats treated with metformin-stimulated Ad-hMSCs showed greater antinociceptive activity and chondroprotective effects than rats treated with unstimulated Ad-hMSCs. This suggests that the immunomodulatory effects of metformin may further enhance the therapeutic effects of MSCs, raising the possibility of clinical application of Ad-hMSCs as a cell-based therapy for OA [136]. A recent study demonstrated that metformin suppresses IL-1β-induced oxidative and OA-like inflammatory changes by stimulating the SIRT3/PINK1/Parkin signaling pathway, which is associated with mitophagy, a process that clears dysfunctional mitochondria [137]. This research highlights a potential use of metformin-mediated mitophagy for the prevention and treatment of OA [138]. Metformin pharmacologic action is affected by organic cation transporters (OCTs) since metformin is an organic cation at physiological pH levels. OCTs are known to mediate metformin entry into target tissues [139,140]. Thus, oral bioavailability and therapeutic efficacy of metformin depend on the transporters [140,141,142]. Further, a diversity of approaches has been proposed and studied to improve the delivery efficacy of metformin for musculoskeletal treatments [143,144,145]. All of the preclinical and clinical data discussed above suggest that metformin is a potential therapeutic strategy for management of OA.

## 7. Conclusions

**Figure 3:**
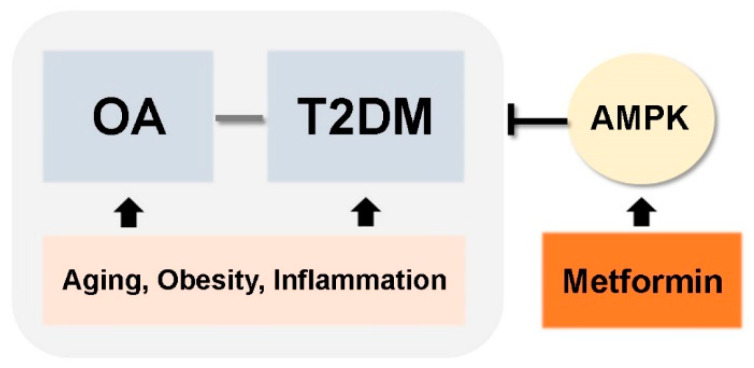
Simplified schematic of therapeutic applications of metformin for OA and T2DM.

In this review, we discussed common risk factors for OA and T2DM, which include aging, obesity, and cytokine- and adipokine-mediated inflammation. These shared pathogenic factors link development of two chronic diseases: OA and T2DM. Accumulated evidence suggests that T2DM could be an independent risk factor for OA development. These findings raise the possibility that medications used to treat T2DM can also be used to treat OA (Figure 3). Among the many different anti-diabetic drugs, we focused on metformin, an activator of AMPK. This is because metformin is most widely used drug in clinical practice, it has a proven and safe pharmacokinetic profile, and it is the mechanisms by which it regulates T2DM are well-known. Currently, several reports demonstrate the potential therapeutic effects of metformin for conditions other than diabetes (e.g., cancer and aging). Although the precise mechanisms by which metformin regulates these other diseases are unclear, the data support further exploration of novel applications. However, further studies are required, and numerous mechanisms must be explained. Here, we discussed the beneficial effects of metformin for the treatment of OA treatment based on data from both preclinical and clinical studies. The data suggest that metformin can be considered as a potential drug for OA. However, we need a better understanding of whether metformin’s beneficial effects on diabetes are reproduced in the context of OA development and progression.
